# Metabolic Syndrome and the Increased Risk of Medically Certified Long-term Sickness Absence: A Prospective Analysis Among Japanese Workers

**DOI:** 10.2188/jea.JE20210185

**Published:** 2023-06-05

**Authors:** Dong V. Hoang, Shamima Akter, Yosuke Inoue, Keisuke Kuwahara, Ami Fukunaga, Zobida Islam, Tohru Nakagawa, Toru Honda, Shuichiro Yamamoto, Hiroko Okazaki, Toshiaki Miyamoto, Takayuki Ogasawara, Naoko Sasaki, Akihiko Uehara, Makoto Yamamoto, Takeshi Kochi, Masafumi Eguchi, Taiki Shirasaka, Makiko Shimizu, Satsue Nagahama, Ai Hori, Teppei Imai, Akiko Nishihara, Kentaro Tomita, Chihiro Nishiura, Maki Konishi, Isamu Kabe, Kenya Yamamoto, Tetsuya Mizoue, Seitaro Dohi

**Affiliations:** 1 Department of Epidemiology and Prevention, National Center for Global Health and Medicine, Tokyo, Japan; 2 Graduate School of Public Health, Teikyo University, Tokyo, Japan; 3 Hitachi Health Care Center, Hitachi, Ltd., Ibaraki, Japan; 4 Mitsui Chemicals, Inc., Tokyo, Japan; 5 Nippon Steel Corporation, East Nippon Works, Chiba, Japan; 6 Mitsubishi Fuso Truck and Bus Corporation, Kanagawa, Japan; 7 Hidaka Tokushukai Hospital, Hokkaido, Japan; 8 Yamaha Corporation, Shizuoka, Japan; 9 Furukawa Electric Co., Ltd., Tokyo, Japan; 10 East Japan Works (Keihin), JFE Steel Corporation, Kanagawa, Japan; 11 All Japan Labour Welfare Foundation, Tokyo, Japan; 12 Department of Global Public Health, Faculty of Medicine, University of Tsukuba, Ibaraki, Japan; 13 OH Support, Kanagawa, Japan; 14 Azbil Corporation, Tokyo, Japan; 15 Healthplant Co., Ltd., Tokyo, Japan; 16 Tokyo Gas Co., Ltd., Tokyo, Japan; 17 Kubota Corporation, Tokyo, Japan; 18 Division of Chemical Information, National Institute of Occupational Safety and Health, Kanagawa, Japan

**Keywords:** sickness absence, metabolic syndrome, longitudinal study, Japan

## Abstract

**Background:**

Metabolic syndrome (MetS) has been associated with various chronic diseases that may lead to long-term sickness absence (LTSA), but there is lacking information on the direct association between MetS and LTSA. The present study aimed to investigate the all-cause and cause-specific associations between MetS and the risk of medically certified LTSA among Japanese workers.

**Methods:**

We recruited 67,403 workers (57,276 men and 10,127 women), aged 20–59 years from 13 companies in Japan during their health check-ups in 2011 (11 companies) and 2014 (2 companies), and we followed them for LTSA events (≥30 consecutive days) until March 31, 2020. MetS was defined according to the Joint Interim Statement. A Cox proportional hazards regression model was used to estimate hazard ratios (HRs) and its 95% confidence intervals (CIs) for LTSA associated with MetS and its components.

**Results:**

During 408,324 person-years of follow-up, 2,915 workers experienced LTSA. The adjusted HR for all-cause LTSA was 1.54 (95% CI, 1.41–1.68) among those with MetS compared to those without MetS. In cause-specific analysis, HRs associated with MetS significantly increased for LTSA due to overall physical disorders (1.76); cardiovascular diseases (3.16); diseases of the musculoskeletal system and connective tissue (2.01); cancers (1.24); obesity-related cancers (1.35); mental, behavioral, and neurodevelopmental disorders (1.28); reaction to severe stress and adjustment disorders (1.46); and external causes (1.46). The number of MetS components were also significantly associated with increased LTSA risk.

**Conclusion:**

MetS was associated with an increase in the risk of LTSA due to various diseases among Japanese workers.

## INTRODUCTION

Sickness absence causes a substantial socio-economic burden for both the employees and their employers.^1^^,^^2^ Annually, workplace absenteeism causes 4–6% loss in gross domestic product in most countries.^3^ Although long-term sickness absence (LTSA) accounts for about one-fifth of all absences, it accounts for 70% of total sickness absence cost.^4^ Identification of modifiable risk factors for LTSA may be of significance to public health. Some examples of such factors are alcohol drinking, smoking,^5^ overtime working hours^6^^,^^7^ and physical inactivity.^8^

Metabolic syndrome (MetS), a clustering of cardiometabolic risk factors,^9^ may be another modifiable risk factor for LTSA. Specifically, MetS is associated with increased risk of various diseases, including cardiovascular disease (CVD),^10^^,^^11^ cancers,^12^ and psychiatric disorders,^13^ which may lead to LTSA.^14^^,^^15^ A few studies have investigated a potentially direct link between MetS and sickness absence.^16^^,^^17^ For example, in a United States study,^16^ workers with MetS had a 31% increase in the odds of experiencing sickness absence (≥3 days). In a recent Japanese study, MetS was associated with an increase in both dental care days and cost.^18^

To date, evidence is lacking on the relationship between MetS and LTSA. Epidemiological data on this issue, including those for specific causes of LTSA and a dose-response relationship, would help occupational physicians protect employees’ health. To address these issues, we prospectively investigated the association of MetS with the risk of all-cause and cause-specific LTSA using data of a large occupational health cohort in Japan. We additionally examined the association between the number of MetS components and LTSA and the association between individual MetS components and LTSA.

## METHODS

### Study setting

The present prospective cohort analysis was based on data of the Japan Epidemiology Collaboration on Occupational Health (J-ECOH) Study, which is an ongoing epidemiological survey investigating health determinants in workers in Japan across various industrial sectors (eg, steel, chemical, gas, plastic product manufacturing, and health care).^19^^–^^21^ We invited companies in the Kanto and Tokai areas in Japan for the study through an occupational physician network (ie, convenience sampling). All the employees for whom the occupational physicians oversaw health management were eligible for the study.

Prior to data collection, the conduct of the J-ECOH Study was announced in each of the participating companies using posters. Specifically, employees were informed that health-related data owned by the company (ie, health check-up, CVD events, LTSA, and death) would be anonymized and provided to the J-ECOH Study, and that one should notify the occupational physician in case of disagreement. This opt-out procedure conforms to the Japanese Ethical Guidelines for Epidemiological Research for observational studies that use existing data. The study protocol was approved by the Ethics Committee of the National Center for Global Health and Medicine, Japan (approval number: NCGM-G-001140).

### Analytic cohort

**Figure 1.**
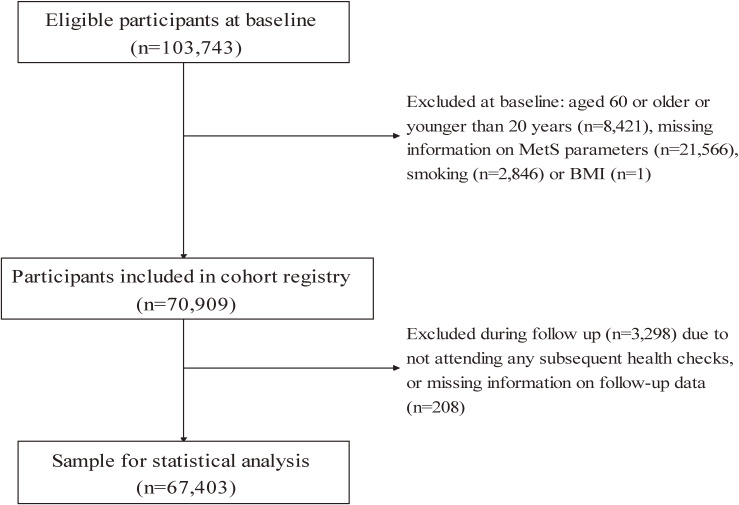
Participants in the Japan Epidemiology Collaboration on Occupational Health Study (*n* = 67,403), Kanto and Tokai, Japan, FY2012-2020. The baseline health check-up was conducted in FY2011, and the follow up period was from April 1, 2012 to March 31, 2020. MetS, metabolic syndrome; FY, Japanese fiscal year, starting from April 1 to March 31 of the following year.

We used information collected from 13 companies which provided health check-up data of the fiscal years of 2011 (11 companies) and 2014 (2 companies). Of the 103,743 individuals (86,747 men and 16,996 women) who had baseline health check-up information, we excluded those aged ≥60 years (the age of retirement; *n* = 6,919) and those aged <20 years (the legal smoking age; *n* = 1,505), as well as those with missing information on MetS parameters (*n* = 21,566), smoking history (*n* = 2,846) and height (*n* = 1). We further excluded workers who did not attend any subsequent health check-ups (*n* = 3,506), leaving 67,403 participants (57,276 men and 10,127 women) for analysis (Figure 1). Those who were excluded were younger, smoked less, drank less alcohol, engaged less in overtime working, and had lower prevalence of comorbidity (psychiatric disorders, CVD, or cancer), but had higher prevalence of MetS, slept longer hours, and were more physically active (eTable 1).

### Health check-up

According to the Industrial Safety and Health Act in Japan, employers must provide employees an opportunity for health check-up, including anthropometric measurements, physical examinations, laboratory tests, and response to a self-administered questionnaire on medical history and lifestyle.

The fasting plasma glucose (FPG) level was measured using either the enzymatic or glucose oxidase peroxidative electrode method. The levels of triglycerides (TG) and high-density lipoprotein cholesterol (HDL-C) were measured using the enzymatic method. Waist circumference (WC) was measured at the umbilical level using a measuring tape, with the subjects in the standing position. Blood pressure (BP) was measured in a sitting position by trained nurses using either an automatic or mercury sphygmomanometer. As the study companies employed different criteria for deciding whether to measure BP twice, we used information on BP collected in the first measurement except for those working for one company which provided only the lower of two measurements.

### Metabolic syndrome and covariates

MetS was defined, according to the Joint Interim Statement^9^ as a clustering of any three or more of the following components: high FPG (≥100 mg/dL or using antidiabetic medication), central obesity (WC ≥90 cm for men or ≥80 cm for women), high TG (≥150 mg/dL or using lipid lowering medication), high BP (systolic BP ≥130 mm Hg, diastolic BP ≥85 mm Hg, or using antihypertensive medication) and low HDL-C (<40 mg/dL for men or <50 mg/dL for women). The cut-off values for WC were based on the recommendation of the World Health Organization for Asian populations.^22^

We selected covariates based on epidemiological evidence for their association with sickness absence: age,^23^ sex,^23^ smoking,^5^ alcohol drinking,^5^ sleep duration,^24^ overtime working hours,^6^ physical activity,^8^ and pre-existing conditions of cancers, psychiatric disorders, and CVDs.^14^^,^^15^ The information on age, sex, smoking status, and pre-existing conditions of cancers, psychiatric disorders, and CVDs was available from all participating companies, while the information on the other covariates was available only in the largest participating company.

### Sickness absence system and registry

In Japan, paid sickness absence is not stipulated by law, and sickness absence schemes vary across individual companies. In the companies participating in the J-ECOH Study, employees were entitled to paid sick-leave with over two-thirds of the salary for at least 18 months, and job security was guaranteed for at least 30 months.^25^ Workers submit a medical certificate written by registered medical doctors when applying for a paid sickness absence.

In April 2012, a within-study registry was created to collect information on LTSA (dates of leave and return and diagnoses of causative disease), cardiovascular events, and death. The information was reported by our collaborating occupational physicians at each company.

### Definition of LTSA

Although LTSA has no universal definition, it is commonly defined as an absence of at least 4 weeks.^26^ In the present study, we defined LTSA as a medically certified sickness absence of ≥30 consecutive days, as all the companies were able to provide the information. We coded the primary causative disease of LTSA according to the International Classification of Diseases, 10^th^ Revision (ICD-10).^27^

The primary outcome of this study was the first medically certified LTSA reported during the follow-up. The secondary outcomes included the first LTSA due to physical disorders (ICD-10 code: A00–B99, C00–D49, D50–D89, E00–E89, G00–G99, H00–H99, I00–I99, J00–J99, K00–K93, L00–L99, M00–M99, N00–N99, O00–O99, Q00–Q99, R00–R99, Z00–Z99); mental, behavioral, and neurodevelopmental disorders (F00–F99); and external causes (S00–T98). The first two groups were further classified into CVDs (I20–I25, I60–I69), diseases of the musculoskeletal system and connective tissue (M00–M99), cancers (C00–D49), obesity-related cancers (cancers of the mouth [C04], pharynx [C14], esophagus [C15], stomach [C16], colon and rectum [C18, C20], liver [C22, including intrahepatic bile duct, C22.1], gallbladder [C23], pancreas [C25], larynx [C32], breast cancer [C50], endometrium [C54], ovary [C56], prostate [C61], and kidney [C64]),^28^ other cancers, depressive episode (F32), and reaction to severe stress and adjustment disorders (F43).

### Statistical analysis

The baseline characteristics of study participants, stratified by MetS status, were described as means and standard deviations (SDs) for continuous variables, and percentages for categorical variables. Person-time was calculated from April 1, 2012 (11 companies) and April 1, 2015 (2 company) to the starting date of the first LTSA episode or the date of censoring, which was determined individually based on available information on annual health check-up, death, retirement, or the end of follow-up (for most companies, this date was the March 31, 2020).

Multilevel Cox proportional hazards regression, in which company was treated as cluster, was used to estimate the hazard ratios (HRs) and 95% confidence intervals (CIs) for all-cause and cause-specific LTSA for participants with versus without MetS. The main analysis consisted of two models: model 1 adjusted for age and sex, and model 2 further adjusted for smoking status (never-smoker, former smoker, or current smoker) and pre-existing conditions of cancer, psychiatric disorders, or cardiovascular diseases (yes or no). For each cause-specific analysis (eg, cancers), those who took LTSA due to other causes were censored on the first date of LTSA. In addition, we conducted analyses stratified by sex.

We also examined the association of the number of and individual MetS components with LTSA. Trend association between the number of MetS components and LTSA was assessed by assigning an ordinal number (1 to 6) to each group, which was treated as a continuous variable when fitted in regression models.

We conducted a series of sensitivity analysis. First, we further adjusted for several other health-related lifestyles that were only available in one of the study companies (*n* = 30,108). More specifically, we adjusted for alcohol consumption (<23 or ≥23 g ethanol/day), sleep duration (<6, 6 to <7, or ≥7 hours/day), overtime working hours (<45, 45 to <80, or ≥80 hours/month), occupational physical activity (mostly sitting, mostly standing or walking, or fairly active) and leisure-time physical activity (<150 or ≥150 min/week) in addition to covariates in model 2, while smoking status was replaced with the information on smoking intensity (never-smoker, former smoker, current smoker consuming 1–10, 11–20, or ≥21 cigarettes per day). Second, we excluded those with pre-existing cancer, CVDs, and psychiatric disorders. Third, we accounted for competing risk events (deaths occurring without any LTSA) using the Fine and Gray method.^29^ Finally, we repeated the main analyses with MetS defined using Japanese criteria (ie, central obesity [WC ≥85 cm in men or ≥90 cm in women] plus two or more of the following: (1) systolic BP ≥130 or diastolic BP ≥85 mm Hg or using antihypertensive medication; (2) FPG ≥110 mg/dL or using antidiabetic medication; and (3) TG ≥150 mg/dL or HDL-C <40 mg/dL).^30^ Statistical significance was set at *P* < 0.05 (two-tailed). All statistical analyses were conducted in RStudio (version 3.2.4; RStudio, PBC, Boston, MA, the United States) using the package “survival” (version 3.1.8).^31^

## RESULTS

Table 1 presents the baseline characteristics of study participants according to MetS status. Compared with participants who did not have MetS, those with MetS were older, more likely to be men, overweight or obese, and former or current smokers; and had higher prevalence of pre-existing conditions of cancer, CVD, and psychiatric disease. In a sub-cohort of workers in the largest company, those with MetS tended to be alcohol drinkers and heavy smokers and to be physically inactive at work and in leisure compared with those without MetS.

**Table 1.** Baseline characteristics of study participants

Characteristics	All participants	MetS status	

		Yes	No
**All companies**			
*N*	67,403	11,630	55,773
Age, mean [SD]	44.6 [8.8]	48.2 [7.4]	43.9 [8.9]
Sex, men	57,276 (85.0)	10,743 (92.4)	46,533 (83.4)
Body mass index, kg/m^2^			
<18.5	3,059 (4.5)	25 (0.2)	3,034 (5.4)
18.5–24.9	45,265 (67.2)	3,043 (26.2)	42,222 (75.7)
25.0–29.9	15,965 (23.7)	6,359 (54.7)	9,606 (17.3)
≥30.0	3,114 (4.6)	2,203 (18.9)	911 (1.6)
Smoking status			
Never-smoker	27,336 (40.6)	3,587 (30.8)	23,749 (42.6)
Former smoker	17,669 (26.2)	3,514 (30.3)	14,155 (25.4)
Current smoker	22,398 (33.2)	4,529 (38.9)	17,869 (32.0)
Pre-existing conditions^a^	2,247 (3.3)	616 (5.3)	1,631 (2.9)
MetS components			
High BP^b^	22,788 (33.8)	9,270 (79.7)	13,518 (24.2)
High FPG^c^	23,017 (34.1)	9,001 (77.4)	14,016 (25.1)
High TG^d^	18,953 (28.1)	9,208 (79.2)	9,745 (17.5)
Central obesity^e^	15,893 (23.6)	8,477 (72.9)	7,416 (13.3)
Low HDL-C^f^	5,377 (8.0)	3,279 (28.2)	2,098 (3.8)
Number of MetS components			
0	22,519 (33.4)	—	22,519 (40.4)
1	19,715 (29.3)	—	19,715 (35.3)
2	13,539 (20.1)	—	13,539 (24.3)
3	7,842 (11.6)	7,842 (67.4)	—
4	3,231 (4.8)	3,231 (27.8)	—
5	557 (0.83)	557 (4.8)	—
**Largest company**			
*N*	30,108	6,354	23,754
Alcohol consumption, g ethanol/day			
<23	22,108 (73.4)	4,481 (70.5)	17,627 (74.2)
≥23	8,000 (26.6)	1,873 (29.5)	6,127 (25.8)
Smoking intensity			
Never-smoker	12,336 (41.0)	2,091 (32.9)	10,245 (43.1)
Former smoker	6,803 (22.6)	1,729 (27.2)	5,074 (21.4)
Current smoker, cigarettes smoked/day			
1–10	2,448 (8.1)	462 (7.3)	1,986 (8.3)
11–20	7,579 (25.2)	1,769 (27.8)	5,810 (24.5)
≥21	942 (3.1)	303 (4.8)	639 (2.7)
Sleeping duration, hours/day			
<6	17,177 (57.1)	3,653 (57.5)	13,524 (56.9)
6 to <7	10,811 (35.9)	2,228 (35.1)	8,583 (36.1)
≥7	2,120 (7.0)	473 (7.4)	1,647 (6.9)
Overtime working, hours/month			
<45	21,187 (70.4)	4,600 (72.4)	16,587 (69.8)
45 to <80	7,653 (25.4)	1,526 (24.0)	6,127 (25.8)
≥80	1,268 (4.2)	228 (3.6)	1,040 (4.4)
Occupational physical activity, operation type			
Mostly sitting	18,984 (63.1)	4,246 (66.8)	14,738 (62.0)
Mostly standing or walking	9,057 (30.0)	1,770 (27.9)	7,287 (30.7)
Fairly active	2,067 (6.9)	338 (5.3)	1,729 (7.3)
Leisure-time physical activity, minutes/week			
<150	26,289 (87.3)	5,569 (87.6)	20,720 (87.2)
≥150	3,819 (12.7)	785 (12.4)	3,034 (12.8)

BP, blood pressure; FPG, fasting plasma glucose; HDL-C, high-density lipoprotein cholesterol; MetS, metabolic syndrome; TG, triglycerides; SD, standard deviation.

Figures in the table are *n* (%), unless otherwise stated.

^a^Cancer, psychiatric and cardiovascular diseases.

^b^Systolic blood pressure ≥130 mm Hg or diastolic blood pressure ≥85 mm Hg or on hypertensive treatment.

^c^Fasting plasma glucose ≥100 mg/dL or on diabetic treatment.

^d^Triglyceride ≥150 mg/dL or on treatment for elevated TG.

^e^Waist circumference ≥90 cm (for men) or ≥80 cm (for women).

^f^High-density lipoprotein cholesterol <40 mg/dL (for men) or <50 mg/dL (for women).

During 408,324 person-years of follow-up, 2,915 participants (2,433 men and 482 women) experienced LTSA. The overall incidence rate of LTSA per 1,000 person-years was 7.10 (95% CI, 6.90–7.40). The specific causes of LTSA are shown in eTable 2.

Table 2 presents the association of MetS with all-cause and cause-specific LTSA. Participants with MetS showed a significantly higher risk of overall LTSA compared with those without MetS; the adjusted HR was 1.54 (95% CI, 1.41–1.68). Regarding the cause-specific LTSA, MetS was associated with increased risk of LTSA due to physical disorders (HR 1.76; 95% CI, 1.56–1.98), CVDs (HR 3.16; 95% CI, 2.35–4.25); diseases of the musculoskeletal system and connective tissue (HR 2.01; 95% CI, 1.54–2.63); cancers (HR 1.24; 95% CI, 1.00–1.53); obesity-related cancers (HR 1.35; 95% CI, 1.00–1.81); mental, behavioral, and neurodevelopmental disorders (HR 1.28; 95% CI, 1.10–1.49); reaction to severe stress and adjustment disorders (HR 1.46; 95% CI, 1.02–2.09), and external causes (HR 1.46; 95% CI, 1.10–1.93). MetS was not associated with LTSA due to depressive episode, which accounted for 60.9% of all mental, behavioral, and neurodevelopmental disorders. There was no measurable difference in association between men and women (eTable 3); The respective HR for all-cause LTSA in men and women was 1.54 (95% CI, 1.41–1.69) and 1.53 (95% CI, 1.15–2.03). HRs for cause-specific LTSA were also similar, while their CIs were much wider in women due to the small sample size.

**Table 2.** Hazard ratios and 95% confidence intervals for medically certified LTSA associated with MetS among Japanese workers

LTSA causes	Hazard ratio (95% confidence interval)^a^			

	All companies		Largest company	

	MetS (−)	MetS (+)	MetS (−)	MetS (+)
*N*	55,773	11,630	23,754	6,354
Person-years	340,252	68,072	159,650	41,385
**All-causes**				
Number of events	2,189	726	870	356
Model 1	1.00 (ref)	1.62 (1.49, 1.77)	1.00 (ref)	1.60 (1.41, 1.81)
Model 2	1.00 (ref)	1.54 (1.41, 1.68)	1.00 (ref)	1.52 (1.34, 1.73)
Model 3	—	—	1.00 (ref)	1.49 (1.31, 1.69)
**Physical disorders**				
Number of events	1,053	428	412	207
Model 1	1.00 (ref)	1.83 (1.62, 2.05)	1.00 (ref)	1.84 (1.55, 2.18)
Model 2	1.00 (ref)	1.76 (1.56, 1.98)	1.00 (ref)	1.78 (1.50, 2.12)
Model 3	—	—	1.00 (ref)	1.76 (1.48, 2.09)
Cardiovascular diseases				
Number of events	101	88	41	50
Model 1	1.00 (ref)	3.40 (2.53, 4.57)	1.00 (ref)	4.00 (2.62, 6.09)
Model 2	1.00 (ref)	3.16 (2.35, 4.25)	1.00 (ref)	3.84 (2.51, 5.86)
Model 3	—	—	1.00 (ref)	3.83 (2.51, 5.86)
Diseases of the musculoskeletal system and connective tissue				
Number of events	186	86	65	48
Model 1	1.00 (ref)	2.09 (1.60, 2.72)	1.00 (ref)	2.83 (1.93, 4.16)
Model 2	1.00 (ref)	2.01 (1.54, 2.63)	1.00 (ref)	2.73 (1.85, 4.02)
Model 3	—	—	1.00 (ref)	2.68 (1.82, 3.95)
Cancers				
Number of events	386	115	171	50
Model 1	1.00 (ref)	1.27 (1.03, 1.57)	1.00 (ref)	1.07 (0.77, 1.47)
Model 2	1.00 (ref)	1.24 (1.00, 1.53)	1.00 (ref)	1.04 (0.75, 1.43)
Model 3	—	—	1.00 (ref)	1.05 (0.76, 1.44)
*Obesity-related cancers*				
Number of events	186	61	73	23
Model 1	1.00 (ref)	1.37 (1.02, 1.84)	1.00 (ref)	1.11 (0.69, 1.79)
Model 2	1.00 (ref)	1.35 (1.00, 1.81)	1.00 (ref)	1.09 (0.68, 1.76)
Model 3	—	—	1.00 (ref)	1.09 (0.68, 1.77)
*Other cancers*				
Number of events	200	54	98	27
Model 1	1.00 (ref)	1.18 (0.87, 1.60)	1.00 (ref)	1.03 (0.67, 1.59)
Model 2	1.00 (ref)	1.14 (0.83, 1.55)	1.00 (ref)	0.99 (0.64, 1.53)
Model 3	—	—	1.00 (ref)	1.01 (0.65, 1.56)
**Mental, behavioral, and neurodevelopmental disorders**				
Number of events	907	225	383	112
Model 1	1.00 (ref)	1.38 (1.19, 1.61)	1.00 (ref)	1.24 (1.00, 1.54)
Model 2	1.00 (ref)	1.28 (1.10, 1.49)	1.00 (ref)	1.14 (0.92, 1.42)
Model 3	—	—	1.00 (ref)	1.10 (0.89, 1.37)
Depressive episode				
Number of events	565	124	248	62
Model 1	1.00 (ref)	1.18 (0.96, 1.44)	1.00 (ref)	1.04 (0.79, 1.39)
Model 2	1.00 (ref)	1.08 (0.88, 1.32)	1.00 (ref)	0.96 (0.72, 1.27)
Model 3	—	—	1.00 (ref)	0.93 (0.70, 1.23)
Reaction to severe stress and adjustment disorders				
Number of events	160	40	68	18
Model 1	1.00 (ref)	1.53 (1.07, 2.19)	1.00 (ref)	1.14 (0.67, 1.93)
Model 2	1.00 (ref)	1.46 (1.02, 2.09)	1.00 (ref)	1.08 (0.64, 1.84)
Model 3	—	—	1.00 (ref)	1.03 (0.60, 1.75)
**External causes**				
Number of events	216	69	75	37
Model 1	1.00 (ref)	1.50 (1.13, 1.99)	1.00 (ref)	1.95 (1.30, 2.92)
Model 2	1.00 (ref)	1.46 (1.10, 1.93)	1.00 (ref)	1.94 (1.29, 2.91)
Model 3	—	—	1.00 (ref)	1.87 (1.25, 2.81)

MetS, metabolic syndrome; LTSA, long-term sickness absence (≥30 consecutive days); ref, reference.

^a^Estimated from multilevel Cox regression (clustered by company); model 1, adjusted for age and sex; model 2, further adjusted for smoking status (never-smoker, former smoker, or current smoker) and pre-existing conditions of cancer, psychiatric and cardiovascular diseases (yes or no); model 3, adjusted for alcohol consumption (<23 or ≥23 g ethanol/day), duration of sleep (<6, 6 to <7, or ≥7 hours/day), overtime working hours (<45, 45 to <80, or ≥80 hours/month), occupational physical activity (mostly sitting, mostly standing or walking, fairly active) and leisure-time physical activity (<150, or ≥150 min/week) plus the covariates included in model 2, while smoking status was replaced with smoking intensity (never-smoker, former smoker, current smoker consuming 1–10, 11–20 or ≥21 cigarette/day).

**Figure 2.**
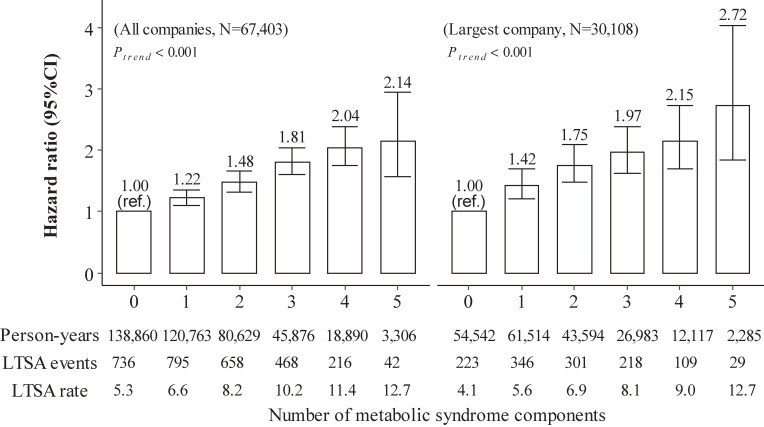
Multivariable-adjusted hazard ratios for the incidence of long-term sickness absence associated with the number of metabolic syndrome components among Japanese workers (*n* = 67,403), Kanto and Tokai, Japan, FY2012–2020. For all companies, Cox regression model was adjusted for age (year), sex (men or women), smoking status (never-smoker, former smoker, or current smoker) and pre-existing conditions of either cancer, psychiatric and cardiovascular diseases (yes or no); For the largest company, Cox regression model was adjusted for alcohol consumption level (<23 or ≥23 g ethanol/day), duration of sleep (<6, 6 to <7, or ≥7 hours/day), overtime working hours (<45, 45 to <80, or ≥80 hours/month), occupational physical activity (mostly sitting, mostly standing or walking, or fairly active) and leisure-time physical activity (<150 or ≥150 min/week), in addition to the covariates included in the model for all companies, while smoking status was replaced with smoking intensity (never-smoker, former smoker, current smoker consuming 1–10, 11–20 or ≥21 cigarette/day). Metabolic syndrome components included high fasting glucose (≥100 mg/dL or on diabetic treatment), central obesity (waist circumference ≥90 cm for men or ≥80 cm for women), high triglyceride (≥150 mg/dL or on treatment for dyslipidemia), elevated blood pressure (systolic blood pressure ≥130 mm Hg or diastolic blood pressure ≥85 mm Hg or on hypertensive treatment) and low high density lipoprotein cholesterol (<40 mg/dL for men or <50 mg/dL for women). LTSA, long-term sickness absence, rate per 1,000 person-years; ref., reference.

Table 3 shows that high FPG, central obesity, high TG, and high BP were each associated with higher LTSA risk; the respective HR was 1.38 (95% CI, 1.27–1.50), 1.22 (95% CI, 1.12–1.33), 1.18 (95% CI, 1.09–1.29) and 1.14 (95% CI, 1.05–1.24) comparing those with versus without the component. Low HDL-C was not associated with LTSA (HR 1.01; 95% CI, 0.89–1.15). The number of MetS components was associated with increased risk of LTSA (Figure 2). Compared with those having no MetS component, the HRs for LTSA among those having 1 to 5 components were 1.22, 1.48, 1.81, 2.04, and 2.14, respectively (*P* for trend <0.001).

**Table 3.** Hazard ratios and 95% confidence intervals for medically certified LTSA associated with each MetS component among Japanese workers

MetS components	Hazard ratio (95% confident interval)^a^			

	All companies		Largest company	

	MetS (−)	MetS (+)	MetS (−)	MetS (+)
**High FPG** ^b^				
*N*	44,386	23,017	16,276	13,832
Person-years	270,791	137,533	109,590	91,445
LTSA events	1,714	1,201	546	680
Model 1	1.00 (ref)	1.49 (1.37, 1.62)	1.00 (ref)	1.56 (1.38, 1.75)
Model 2	1.00 (ref)	1.38 (1.27, 1.50)	1.00 (ref)	1.44 (1.28, 1.62)
Model 3	—	—	1.00 (ref)	1.46 (1.30, 1.65)
**Central obesity** ^c^				
*N*	51,510	15,893	22,450	7,658
Person-years	313,278	95,047	150,506	50,528
LTSA events	2,011	904	813	413
Model 1	1.00 (ref)	1.42 (1.31, 1.53)	1.00 (ref)	1.49 (1.33, 1.68)
Model 2	1.00 (ref)	1.22 (1.12, 1.33)	1.00 (ref)	1.27 (1.12, 1.45)
Model 3	—	—	1.00 (ref)	1.25 (1.10, 1.42)
**High TG** ^d^				
*N*	48,450	18,953	20,810	9,298
Person-years	294,178	114,147	139,436	61,599
LTSA events	1,889	1,026	757	469
Model 1	1.00 (ref)	1.40 (1.29, 1.51)	1.00 (ref)	1.44 (1.28, 1.62)
Model 2	1.00 (ref)	1.18 (1.09, 1.29)	1.00 (ref)	1.22 (1.08, 1.38)
Model 3	—	—	1.00 (ref)	1.21 (1.06, 1.37)
**High BP** ^e^				
*N*	44,615	22,788	20,153	9,955
Person-years	276,483	131,842	136,658	64,376
LTSA events	1,742	1,173	760	466
Model 1	1.00 (ref)	1.28 (1.19, 1.39)	1.00 (ref)	1.31 (1.16, 1.47)
Model 2	1.00 (ref)	1.14 (1.05, 1.24)	1.00 (ref)	1.14 (1.01, 1.30)
Model 3	—	—	1.00 (ref)	1.15 (1.02, 1.31)
**Low HDL-C** ^f^				
*N*	62,026	5,377	26,874	3,234
Person-years	375,153	33,172	179,439	21,596
LTSA events	2,630	285	1,071	155
Model 1	1.00 (ref)	1.25 (1.11, 1.42)	1.00 (ref)	1.20 (1.02, 1.43)
Model 2	1.00 (ref)	1.01 (0.89, 1.15)	1.00 (ref)	0.96 (0.81, 1.15)
Model 3	—	—	1.00 (ref)	0.91 (0.76, 1.08)

BP, blood pressure; FPG, fasting plasma glucose; HDL-C, high-density lipoprotein cholesterol; LTSA, long-term sickness absence (≥30 consecutive days); MetS, metabolic syndrome; ref, reference; TG, triglycerides.

^a^Estimated from multilevel Cox regression (clustered by company); model 1, adjusted for age and sex; model 2, further adjusted for smoking status (never-smoker, former smoker, or current smoker) and pre-existing conditions of cancer, psychiatric and cardiovascular diseases (yes or no); model 3, adjusted for alcohol consumption level (<23 or ≥23 g ethanol/day), duration of sleep (<6, 6 to <7, or ≥7 hours/day), overtime working hours (<45, 45 to <80, or ≥80 hours/month), occupational physical activity (mostly sitting, mostly standing or walking, or fairly active) and leisure-time physical activity (<150 or ≥150 min/week), plus the covariates included in the model 2, while smoking status was replaced with smoking intensity (never-smoker, former smoker, current smoker consuming 1–10, 11–20 or ≥21 cigarette/day). In model 2 and model 3, the association of each MetS component with LTSA was mutually adjusted for the other components.

^b^Fasting plasma glucose ≥100 mg/dL or using antidiabetic medication.

^c^Waist circumference ≥90 cm (for men) or ≥80 cm (for women).

^d^Triglyceride ≥150 mg/dL or using lipid lowering medication.

^e^Systolic blood pressure ≥130 mm Hg or diastolic blood pressure ≥85 mm Hg or using antihypertensive medication.

^f^High density lipoprotein cholesterol <40 mg/dL (for men) or <50 mg/dL (for women).

A series of sensitivity analyses all reported similar results. For example, after adjusting for additional covariates among participants working for the largest company, the results did not materially change (model 3 in Table 2 and Table 3). When we excluded those with baseline cancer, CVDs, and psychiatric disorders (eTable 4), there remained significant associations, albeit with slightly reduced magnitudes for LTSA due to CVDs (HR 3.03) and reaction to severe stress and adjustment disorders (HR 1.32). After accounting for death events, the point estimates for the MetS-LTSA association remained almost the same (HR 1.54; 95% CI, 1.41–1.68) (eTable 5). With the MetS defined according to the Japanese criteria, the association became more apparent for LTSA due to physical disorders (HR 1.98), cancer (HR 1.60), and obesity-related cancers (HR 1.78) (eTable 6).

## DISCUSSION

In the present large-scale prospective cohort study among Japanese workers, MetS was associated with higher risk of LTSA, especially LTSA due to CVD. The association between MetS and LTSA was similar for men and women. The individual components of MetS, except for low HDL-C (ie, high FBG, central obesity, high TG, and high BP), were associated with increased LTSA risk. When we examined the association between the number of MetS components and the risk of LTSA, we observed a dose-response relationship.

While we are not aware of any study that had specifically examined the association between MetS and LTSA, this study is in line with a United States study,^16^ which reported that MetS and high TG were significantly associated with sickness absence (≥3 days). In addition, in a Finnish study,^32^ central obesity was significantly associated with increased risk of sickness absence in both men (relative risk [RR] 1.63) and women (RR 1.89). The present study extends the previous observations by showing the association between MetS and LTSA, which was similar for men and women. We also found a dose-response relationship between the number of MetS components and LTSA risk, which highlights the importance of studying MetS as a whole, instead of individual components.

The present finding of a significant association between MetS and LTSA due to physical disorders agrees with epidemiological data linking MetS to the risk of CVDs, cancers,^33^ and diseases of musculoskeletal system,^34^^,^^35^ which are major causes of LTSA.^15^^,^^36^ Of these, the link between MetS and CVDs has been well established. In a meta-analysis of 87 studies,^10^ MetS was associated with increased risk of CVDs (HR 2.35), myocardial infarction (RR 1.99), and stroke (RR 2.27). Regarding neoplasm, a systematic review and meta-analysis of 43 studies^12^ demonstrated links of MetS to obesity-related cancers, including those of the liver (RR 1.43 in men), pancreas (RR 1.58 in women), and colon and rectum (RR 1.25 in men, 1.34 in women). As for diseases of musculoskeletal system, MetS was associated with increased risk of osteoporosis in men^34^ and osteoarthritis in women.^35^

MetS was also associated with increased risk of LTSA due to mental, behavioral, and neurodevelopmental disorders. In the sub-analysis on major diseases within this category, we found no association in relation to depressive episode but observed significant association between MetS and LTSA due to reaction to severe stress and adjustment disorders. Our null finding in relation to depressive episode seems to contradict those of a meta-analysis of 9 cohort studies showing a significant association between MetS and depression.^13^ This discrepancy might have resulted from a difference in the cardiometabolic risk profile between this population and those included in the meta-analysis; more specifically, central obesity, which showed a pronounced association in the meta-analysis, was present in 23.6% of our study population, while high BP and high FPG, which were not significantly associated with depression in the meta-analysis, were more common in this study (33.8% and 34.1%, respectively). As for the significant association between MetS and LTSA due to reaction to severe stress and adjustment disorders, we are not aware of epidemiological evidence that supports or refutes the finding, which requires confirmation in future studies. Nevertheless, the observed association between MetS and mental, behavioral, and neurodevelopmental disorders signifies the importance of integrating psychological assessment and intervention in the prevention of LTSA for those with MetS.

The present association between MetS and LTSA due to external causes, which mainly consisted of fractures (49.0%), contradicts previous studies that showed significant associations only among women but not among men, given that approximately 85% of our study participants were male. For example, a cross-sectional study in China^37^ found that MetS was associated with an increased likelihood of having recent history of osteoporotic fracture in women (odds ratio 1.22). A few other studies in China^38^^,^^39^ and the United States^40^ also reported an association of MetS with increased risk of fracture in women, but not in men. It should be also of note, however, that a meta-analysis of 9 studies showed that MetS was associated with lower bone mineral density,^34^ which might explain the observed MetS-fracture association.

Despite epidemiological evidence for the association of MetS with various causes of LTSA,^10^^,^^12^^,^^13^^,^^41^ the mechanisms underlying these links are complicated and have not been fully understood.^33^ For example, excessive adipocytokines secreted by abdominal fat could result in both microvascular injuries and increased cell proliferation, which respectively lead to CVDs^33^ and cancer progression.^42^ This inflammatory-mediating mechanism may also underlie etiological pathway of several musculoskeletal diseases (eg, osteoporosis).^43^ Differently, the comorbidity of MetS with psychiatric disorders is regulated by other mechanisms. For instance, a hyperactivation of the hypothalamic-pituitary-adrenal axis would result in both high plasma TG and depression.^13^

The robustness of the present association between MetS and LTSA was confirmed through a series of sensitivity analyses. First, our sensitivity analyses in which we adjusted for additional covariates in the largest company (model 3) showed a similar pattern of the associations of MetS and its components with LTSA risk compared to those observed in model 2. When we excluded participants with baseline cancer, CVDs, and psychiatric disorders from the analyses, the results did not materially change. While these conditions can be regarded as mediators in the association between MetS and LTSA^14^^,^^15^^,^^44^ (and thus we did not exclude such participants in the main analysis), this sensitivity analysis confirms the strong association between MetS and LTSA. This association also remained almost unchanged after accounting for death events occurring without LTSA.

In Japan, the Specific Health Checkups and Specific Health Guidance have been widely implemented to mitigate the burden of lifestyle diseases.^45^ This program was designed to identify those with MetS, followed by health guidance toward healthy lifestyle, including weight control and no smoking. The present findings on MetS, together with previous ones on obesity^20^ and smoking^21^ in relation to LTSA in J-ECOH study, imply that such comprehensive programs may contribute to the reduction of the burden of LTSA due to various diseases.

The present findings must be interpreted with several limitations. First, as we do not have detailed information on the exact number of employees working for the participating companies or those who refused to participate in survey, we were unable to calculate exact participation or refusal rates. However, it is likely that the participation rate was higher than 95% (because employers are required by law to organize annual health check-ups) and the refusal rate was less than 1%. Second, we treated workers with sickness absence of less than 30 consecutive days as those who never experienced sickness absence. This might have attenuated the association of MetS with increased LTSA risk. The observed association might also have been attenuated by a tendency to not take LTSA among workers who were about to retire, or confounded by previous LTSA, socio-economic status, and other factors (eg, employment types [full-time, part-time, contract and temporary workers] and the availability of worksite health promotion activities). Third, our convenient recruitment of participating companies might also lead to selection bias. Finally, since the study population was mainly comprised of men (85%) working for private companies, caution should be exercised when generalizing the findings.

In conclusion, the presence of MetS or at least one of its components (except for low HDL-C) was associated with a higher risk of LTSA among Japanese workers. Furthermore, LTSA risk increased with increasing number of MetS components.

## SUPPLEMENTARY MATERIAL

eTable 1. Baseline characteristics of eligible participants

eTable 2. Medically certified causes for which participants took LTSA

eTable 3. Hazard ratios and 95% confidence intervals for LTSA associated with MetS among Japanese workers, stratified by sex

eTable 4. Hazard ratios and 95% confidence intervals for LTSA associated with MetS in Japanese workers, excluding those with baseline, CVDs or psychiatric disorders

eTable 5. Hazard ratios and 95% confidence intervals for all-cause LTSA associated with MetS in Japanese workers, accounting for competing death events

eTable 6. Hazard ratios and 95% confidence intervals for LTSA associated with MetS in Japanese workers, with MetS defined according to Japanese criteria
