# Supplementary material for: Metabolic Syndrome and the Increased Risk of Medically Certified Long-term Sickness Absence: A Prospective Analysis Among Japanese Workers
Source: J Epidemiol. 2023 Jun 5;33(6):311–20. doi: 10.2188/jea.JE20210185 (PMC10165215; doi:10.2188/jea.JE20210185)
Supplement: Supplementary file 1 [file je-33-311-s001.pdf]

eTable 1. Baseline characteristics of eligible participants

Characteristics	Individuals included in the current study	Individuals excluded from the current study
**All companies**		
N	67,403	36,340
Age, years, mean [SD]	44.6 [8.8]	38.5 [15.0]
Sex, men	57,276 (85.0)	29,471 (81.1)
Body mass index, kg/m^2 [a]		
<18.5	3,059 (4.5)	2,270 (6.3)
18.5–24.9	45,265 (67.2)	25,020 (69.3)
25.0–29.9	15,965 (23.7)	7,272 (20.1)
≥30.0	3,114 (4.6)	1,567 (4.3)
Smoking status [b]		
Never smoker	27,336 (40.6)	13,691 (46.1)
Former smoker	17,669 (26.2)	6,959 (23.5)
Current smoker	22,398 (33.2)	9,020 (30.4)
Metabolic syndrome status [c]	11,630 (17.3)	3,116 (22.5)
Psychiatric disorders	1,057 (1.6)	325 (0.89)
Cardiovascular diseases	720 (1.1)	304 (0.84)
Cancers	510 (0.76)	219 (0.60)
Other chronic diseases		
Hypertension	5,536 (8.2)	2,517 (6.9)
Diabetes	1,960 (2.9)	904 (2.5)
Lipidaemia	2,867 (4.3)	941 (2.6)
Gout or hyperuricemia	1,541 (2.3)	499 (1.4)
Anaemia	373 (0.55)	198 (0.54)
Chronic hepatitis	116 (0.17)	48 (0.13)
Liver diseases (excluding fatty liver)	27 (0.04)	14 (0.04)
Thyroid diseases	284 (0.42)	104 (0.29)
Asthma	542 (0.80)	373 (1.03)
Kidney diseases	142 (0.21)	85 (0.23)
Pancreatic diseases	11 (0.02)	6 (0.02)
**Largest company**		
N	30,108	24,788
Alcohol consumption, g ethanol/week		
<23	22,108 (73.4)	21,925 (88.5)
≥23	8,000 (26.6)	2,863 (11.5)
Smoking intensity [d]		
Never-smoker	12,336 (41.0)	10,453 (54.1)
Former smoker	6,803 (22.6)	2,935 (15.2)
Current smoker, cigarettes smoked/day		
1–10	2,448 (8.1)	1,965 (10.2)
11–20	7,579 (25.2)	3,594 (18.6)
≥21	942 (3.1)	364 (1.9)
Sleeping duration, hours/day [e]		
<6	17,177 (57.1)	9,437 (50.1)
6–7	10,811 (35.9)	7,494 (39.7)
>7	2,120 (7.0)	1,923 (10.2)

Overtime working, hours/month		
<45	21,187 (70.4)	21,081 (85.1)
45 to <80	7,653 (25.4)	3,280 (13.2)
≥80	1,268 (4.2)	427 (1.7)
Occupational physical activity, operation type [f]		
Almost sitting	18,984 (63.1)	10,988 (56.8)
Mostly standing or walking	9,057 (30.1)	6,693 (34.6)
Fairly active	2,067 (6.8)	1,665 (8.6)
Leisure-time physical activity, minutes/week [g]		
<150	26,289 (87.3)	15,124 (84.6)
≥150	3,819 (12.7)	2,747 (15.4)

SD, standard deviation.
Figures in the table are n (%), unless otherwise stated.
[a] excluding 211 with missing information
[b] excluding 6,670 with missing information
[c] excluding 22,471with missing information
[d] excluding 5,477 with missing information
[e] excluding 5,934 with missing information
[f] excluding 5,442 with missing information
[g] excluding 6,917 with missing information

eTable 2. Medically certified causes for which participants took LTSA

Medically certified causes	ICD-10	N
**Physical disorders**		**1,481**
Cancers	C00–D49	501
Diseases of the circulatory system	I00–I99	288
Diseases of the musculoskeletal system and connective tissue	M00–M99	272
Diseases of the nervous system	G00–G99	98
Diseases of the digestive system	K00–K93	88
Endocrine, nutritional and metabolic diseases	E00–E89	42
Diseases of the genitourinary system	N00–N99	30
Diseases of the respiratory system	J00–J99	25
Diseases of the eye and adnexa	H00–H59	25
Pregnancy, childbirth and the puerperium	O00–O99	24
Certain infectious and parasitic diseases	A00–B99	21
Others		67
**Mental, behavioral, and neurodevelopmental disorders**		**1,132**
Depressive episode	F32	689
Reaction to severe stress and adjustment disorders	F43	200
Other anxiety disorders	F41	59
Bipolar affective disorder	F31	31
Somatoform disorders	F45	27
Schizophrenia	F20	24
Other neurotic disorders	F48	22
Others		80
**External causes**		**285**
Fractures	S32, S42, S52, S72, S82, S92	140
Injuries	S46, S86	30
Others		115
**Not available**		17
**Total**		**2,915**

ICD, International classification of disease; LTSA, long-term sickness absence.

eTable 3. Hazard ratios and 95% confidence intervals for LTSA associated with MetS among Japanese workers, stratified by sex

LTSA causes	Hazard ratio (95% confidence interval) [a]			
	Men (n=57,276)		Women (n=10,127)	
	MetS (-)	MetS (+)	MetS (-)	MetS (+)
N	46,533	10,743	9,240	887
Person-years	286,997	63,086	53,255	4,986
**All-cause LTSA**				
Number of events	1766	667	423	59
Model 1	1.00 (ref)	1.63 (1.49, 1.78)	1.00 (ref)	1.58 (1.19, 2.09)
Model 2	1.00 (ref)	1.54 (1.41, 1.69)	1.00 (ref)	1.53 (1.15, 2.03)
**Physical disorders**				
Number of events	803	389	250	39
Model 1	1.00 (ref)	1.87 (1.65, 2.11)	1.00 (ref)	1.57 (1.11, 2.23)
Model 2	1.00 (ref)	1.79 (1.58, 2.03)	1.00 (ref)	1.54 (1.08, 2.18)
Cardiovascular diseases				
Number of events	89	85	12	3
Model 1	1.00 (ref)	3.52 (2.59, 4.77)	1.00 (ref)	2.14 (0.58, 7.93)
Model 2	1.00 (ref)	3.28 (2.41, 4.45)	1.00 (ref)	2.10 (0.57, 7.75)
Diseases of the musculoskeletal system and connective tissue				
Number of events	140	76	46	10
Model 1	1.00 (ref)	2.18 (1.63, 2.90)	1.00 (ref)	1.72 (0.85, 3.47)
Model 2	1.00 (ref)	2.07 (1.55, 2.76)	1.00 (ref)	1.76 (0.87, 3.57)
Cancers				
Number of events	289	101	97	14
Model 1	1.00 (ref)	1.25 (1.00, 1.58)	1.00 (ref)	1.36 (0.76, 2.41)
Model 2	1.00 (ref)	1.22 (0.97, 1.54)	1.00 (ref)	1.33 (0.75, 2.36)
Obesity-related cancers				
Number of events	138	53	48	8
Model 1	1.00 (ref)	1.36 (0.98, 1.87)	1.00 (ref)	1.44 (0.67, 3.10)
Model 2	1.00 (ref)	1.34 (0.97, 1.85)	1.00 (ref)	1.41 (0.65, 3.05)
Other cancers				
Number of events	151	48	49	6
Model 1	1.00 (ref)	1.16 (0.84, 1.62)	1.00 (ref)	1.26 (0.53, 3.01)
Model 2	1.00 (ref)	1.12 (0.80, 1.56)	1.00 (ref)	1.22 (0.51, 2.90)
**Mental, behavioral, and neurodevelopmental disorders**				
Number of events	786	216	121	9
Model 1	1.00 (ref)	1.38 (1.19, 1.62)	1.00 (ref)	1.23 (0.62, 2.46)
Model 2	1.00 (ref)	1.28 (1.09, 1.49)	1.00 (ref)	1.17 (0.59, 2.34)
Depressive episode				
Number of events	500	120	65	4
Model 1	1.00 (ref)	1.17 (0.96, 1.44)	1.00 (ref)	1.12 (0.40, 3.14)
Model 2	1.00 (ref)	1.07 (0.87, 1.31)	1.00 (ref)	1.06 (0.38, 2.96)
Reaction to severe stress and adjustment disorders				
Number of events	136	36	24	4

Model 1	1.00 (ref)	1.42 (0.97, 2.08)	1.00 (ref)	3.06 (1.02, 9.21)
Model 2	1.00 (ref)	1.35 (0.92, 1.98)	1.00 (ref)	3.13 (1.03, 9.50)
**External causes**				
Number of events	167	58	49	11
Model 1	1.00 (ref)	1.44 (1.06, 1.96)	1.00 (ref)	1.84 (0.94, 3.62)
Model 2	1.00 (ref)	1.40 (1.03, 1.90)	1.00 (ref)	1.78 (0.91, 3.50)

LTSA, long-term sickness absence (≥30 consecutive days); MetS, metabolic syndrome; ref, reference.

[a] estimated from multilevel Cox regression (clustered by company).

Model 1, adjusted for age and sex. Model 2, further adjusted for smoking status (never-smoker, former smoker, or current smoker) and pre-existing conditions of cancer, psychiatric and cardiovascular diseases (yes or no)

eTable 4. Hazard ratios and 95% confidence intervals for LTSA associated with MetS in Japanese workers, excluding those with baseline, CVDs or psychiatric disorders

LTSA causes	Hazard ratio (95% confidence interval) [a]			
	All companies		Largest company	
	MetS (-)	MetS (+)	MetS (-)	MetS (+)
N	54,142	11,014	22,974	5,961
Person-years	330,978	64,795	154,765	39,097
**All-cause LTSA**				
Number of events	2004	631	796	302
Model 1	1.00 (ref)	1.57 (1.43, 1.72)	1.00 (ref)	1.53 (1.33, 1.75)
Model 2	1.00 (ref)	1.54 (1.40, 1.69)	1.00 (ref)	1.51 (1.32, 1.73)
Model 3	-	-	1.00 (ref)	1.47 (1.29, 1.69)
**Physical disorders**				
Number of events	992	376	389	178
Model 1	1.00 (ref)	1.73 (1.53, 1.96)	1.00 (ref)	1.73 (1.44, 2.07)
Model 2	1.00 (ref)	1.70 (1.50, 1.92)	1.00 (ref)	1.71 (1.43, 2.05)
Model 3	-	-	1.00 (ref)	1.68 (1.40, 2.02)
Cardiovascular diseases				
Number of events	91	72	40	41
Model 1	1.00 (ref)	3.13 (2.28, 4.31)	1.00 (ref)	3.47 (2.22, 5.41)
Model 2	1.00 (ref)	3.03 (2.20, 4.16)	1.00 (ref)	3.43 (2.20, 5.34)
Model 3	-	-	1.00 (ref)	3.46 (2.22, 5.41)
Diseases of the musculoskeletal system and connective tissue				
Number of events	176	78	61	41
Model 1	1.00 (ref)	2.06 (1.56, 2.71)	1.00 (ref)	2.66 (1.77, 4.00)
Model 2	1.00 (ref)	2.02 (1.53, 2.66)	1.00 (ref)	2.65 (1.76, 3.98)
Model 3	-	-	1.00 (ref)	2.54 (1.69, 3.83)
Cancers				
Number of events	364	108	157	48
Model 1	1.00 (ref)	1.29 (1.04, 1.61)	1.00 (ref)	1.15 (0.83, 1.60)
Model 2	1.00 (ref)	1.27 (1.02, 1.58)	1.00 (ref)	1.14 (0.82, 1.58)
Model 3	-	-	1.00 (ref)	1.15 (0.82, 1.60)
Obesity-related cancers				
Number of events	176	56	68	22
Model 1	1.00 (ref)	1.35 (0.99, 1.84)	1.00 (ref)	1.17 (0.72, 1.90)
Model 2	1.00 (ref)	1.34 (0.98, 1.82)	1.00 (ref)	1.16 (0.71, 1.90)
Model 3	-	-	1.00 (ref)	1.17 (0.71, 1.91)
Other cancers				
Number of events	188	52	89	26
Model 1	1.00 (ref)	1.24 (0.90, 1.70)	1.00 (ref)	1.14 (0.73, 1.78)
Model 2	1.00 (ref)	1.21 (0.88, 1.65)	1.00 (ref)	1.12 (0.72, 1.75)
Model 3	-	-	1.00 (ref)	1.13 (0.72, 1.76)
**Mental, behavioral, and neurodevelopmental disorders**				
Number of events	793	186	332	90

Model 1	1.00 (ref)	1.34 (1.14, 1.58)	1.00 (ref)	1.19 (0.94, 1.51)
Model 2	1.00 (ref)	1.32 (1.12, 1.55)	1.00 (ref)	1.17 (0.92, 1.49)
Model 3	-	-	1.00 (ref)	1.14 (0.90, 1.44)
Depressive episode				
Number of events	485	100	212	48
Model 1	1.00 (ref)	1.14 (0.91, 1.42)	1.00 (ref)	0.98 (0.71, 1.35)
Model 2	1.00 (ref)	1.12 (0.90, 1.39)	1.00 (ref)	0.97 (0.70, 1.33)
Model 3	-	-	1.00 (ref)	0.95 (0.69, 1.31)
Reaction to severe stress and adjustment disorders				
Number of events	150	33	65	15
Model 1	1.00 (ref)	1.34 (0.91, 1.98)	1.00 (ref)	1.02 (0.57, 1.80)
Model 2	1.00 (ref)	1.32 (0.89, 1.95)	1.00 (ref)	0.99 (0.56, 1.75)
Model 3	-	-	1.00 (ref)	0.95 (0.53, 1.68)
**External causes**				
Number of events	207	65	75	34
Model 1	1.00 (ref)	1.49 (1.12, 1.99)	1.00 (ref)	1.83 (1.21, 2.78)
Model 2	1.00 (ref)	1.46 (1.10, 1.95)	1.00 (ref)	1.82 (1.20, 2.75)
Model 3	-	-	1.00 (ref)	1.76 (1.16, 2.67)

CVD, cardiovascular diseases; LTSA, long-term sickness absence (≥30 consecutive days); MetS, metabolic syndrome; ref, reference.

[a] estimated from multilevel Cox regression (clustered by company); Model 1, adjusted for age and sex; Model 2, further adjusted for smoking status (never-smoker, former smoker, or current smoker) and pre-existing conditions of cancer, psychiatric and cardiovascular diseases (yes or no); Model 3, adjusted for alcohol consumption (<23 or ≥23 g ethanol/day), duration of sleep (<6, 6 to <7, or ≥7 hours/day), overtime working hours (<45, 45 to <80, or ≥80 hours/month), occupational physical activity (mostly sitting, mostly standing or walking, or fairly active) and leisure-time physical activity (<150 or ≥150 min/week) plus the covariates included in the Model 2, while smoking status was replaced with smoking intensity (never-smoker, former smoker, current smoker consuming 1–10, 11–20 or ≥21 cigarette/day).

eTable 5. Hazard ratios and 95% confidence intervals for all-cause LTSA associated with MetS in Japanese workers, accounting for competing death events

	Sub-distribution hazard ratio (95% confidence interval) [a]			
	All companies		Largest company	
	**MetS (-)**	**MetS (+)**	**MetS (-)**	**MetS (+)**
Number of participants	55,773	11,630	23,754	6,354
Person-years	340,252	68,072	159,650	41,385
Death events	124	49	53	28
**All-cause LTSA**				
LTSA events	2,189	726	870	356
Model 1	1.00 (ref)	1.62 (1.48, 1.77)	1.00 (ref)	1.60 (1.41, 1.81)
Model 2	1.00 (ref)	1.54 (1.41, 1.68)	1.00 (ref)	1.52 (1.34, 1.73)
Model 3	-	-	1.00 (ref)	1.49 (1.31, 1.69)

LTSA, long-term sickness absence (≥ 30 consecutive days); MetS, metabolic syndrome; ref, reference.

[a] estimated using Fine and Gray method (1999); Model 1, adjusted for age (year), sex and company; Model 2, adjusted for smoking status (never-smoker, former smoker, or current smoker), pre-existing conditions of cancer, psychiatric or cardiovascular diseases plus the covariates in the Model 1; Model 3, adjusted for alcohol consumption (<23 or ≥23 g ethanol/day), duration of sleep (<6, 6 to <7, or ≥7 hours/day), overtime working hours (<45, 45 to <80, or ≥80 hours/month), occupational physical activity (mostly sitting, mostly standing or walking, or fairly active) and leisure-time physical activity (<150 or ≥150 min/week) plus the covariates in the Model 2, while smoking status was replaced with smoking intensity (never-smoker, former smoker, current smoker consuming 1–10, 11–20 or ≥21 cigarette/day)

eTable 6. Hazard ratios and 95% confidence intervals for LTSA associated with MetS in Japanese workers, with MetS defined according to Japanese criteria

**LTSA causes**	**Hazard ratio (95% confidence interval)** [a]			
	**All companies**		**Largest company**	
	MetS (-)	MetS (+)	MetS (-)	MetS (+)
N	58,761	8,642	25,736	4,372
Person-years	358,222	50,102	172,740	28,294
**All-cause LTSA**				
Number of events	2348	567	963	263
Model 1	1.00 (ref)	1.68 (1.53, 1.84)	1.00 (ref)	1.72 (1.49, 1.97)
Model 2	1.00 (ref)	1.65 (1.50, 1.82)	1.00 (ref)	1.71 (1.49, 1.97)
Model 3	-	-	1.00 (ref)	1.68 (1.46, 1.93)
**Physical disorders**				
Number of events	1133	348	458	161
Model 1	1.00 (ref)	2.02 (1.78, 2.28)	1.00 (ref)	2.15 (1.78, 2.59)
Model 2	1.00 (ref)	1.98 (1.75, 2.25)	1.00 (ref)	2.16 (1.79, 2.60)
Model 3	-	-	1.00 (ref)	2.14 (1.77, 2.58)
Cardiovascular diseases				
Number of events	120	69	53	38
Model 1	1.00 (ref)	3.09 (2.28, 4.20)	1.00 (ref)	3.72 (2.41, 5.73)
Model 2	1.00 (ref)	3.01 (2.22, 4.08)	1.00 (ref)	3.74 (2.43, 5.76)
Model 3	-	-	1.00 (ref)	3.75 (2.43, 5.79)
Diseases of the musculoskeletal system and connective tissue				
Number of events	206	66	79	34
Model 1	1.00 (ref)	2.15 (1.61, 2.87)	1.00 (ref)	2.92 (1.91, 4.48)
Model 2	1.00 (ref)	2.10 (1.58, 2.81)	1.00 (ref)	2.89 (1.89, 4.43)
Model 3	-	-	1.00 (ref)	2.84 (1.85, 4.36)
Cancers				
Number of events	400	101	175	46
Model 1	1.00 (ref)	1.62 (1.29, 2.03)	1.00 (ref)	1.67 (1.19, 2.33)
Model 2	1.00 (ref)	1.60 (1.27, 2.00)	1.00 (ref)	1.66 (1.19, 2.33)
Model 3	-	-	1.00 (ref)	1.68 (1.20, 2.35)
Obesity-related cancers				
Number of events	192	55	70	26
Model 1	1.00 (ref)	1.79 (1.31, 2.45)	1.00 (ref)	2.39 (1.49, 3.84)
Model 2	1.00 (ref)	1.78 (1.31, 2.43)	1.00 (ref)	2.41 (1.50, 3.86)
Model 3	-	-	1.00 (ref)	2.41 (1.50, 3.87)
Other cancers				
Number of events	208	46	105	20
Model 1	1.00 (ref)	1.46 (1.05, 2.03)	1.00 (ref)	1.20 (0.73, 1.96)
Model 2	1.00 (ref)	1.43 (1.03, 1.99)	1.00 (ref)	1.19 (0.73, 1.94)
Model 3	-	-	1.00 (ref)	1.21 (0.74, 1.98)
**Mental-behavioral and neurodevelopmental disorders**				
Number of events	966	166	415	80
Model 1	1.00 (ref)	1.33 (1.12, 1.57)	1.00 (ref)	1.28 (1.00, 1.63)
Model 2	1.00 (ref)	1.31 (1.11, 1.55)	1.00 (ref)	1.27 (0.99, 1.62)

Model 3	-	-	1.00 (ref)	1.23 (0.96, 1.57)
Depressive episode				
Number of events	597	92	264	46
Model 1	1.00 (ref)	1.15 (0.92, 1.43)	1.00 (ref)	1.13 (0.82, 1.55)
Model 2	1.00 (ref)	1.13 (0.90, 1.42)	1.00 (ref)	1.12 (0.82, 1.54)
Model 3	-	-	1.00 (ref)	1.10 (0.80, 1.51)
Reaction to severe stress and adjustment disorders				
Number of events	169	31	70	16
Model 1	1.00 (ref)	1.56 (1.05, 2.32)	1.00 (ref)	1.54 (0.89, 2.68)
Model 2	1.00 (ref)	1.55 (1.04, 2.29)	1.00 (ref)	1.52 (0.88, 2.65)
Model 3	-	-	1.00 (ref)	1.45 (0.83, 2.52)
**External causes**				
Number of events	236	49	90	22
Model 1	1.00 (ref)	1.40 (1.02, 1.92)	1.00 (ref)	1.60 (0.99, 2.59)
Model 2	1.00 (ref)	1.37 (0.99, 1.88)	1.00 (ref)	1.59 (0.98, 2.57)
Model 3	-	-	1.00 (ref)	1.55 (0.96, 2.52)

LTSA, long-term sickness absence (≥ 30 consecutive days); MetS, metabolic syndrome; ref, reference.
Japanese criteria for MetS: central obesity (waist circumference ≥ 85 cm in men or ≥ 90 cm in women) plus two or more of the following: (1) BP of 130/85mmHg or on treatment of hypertension; (2) FPG ≥110mg/dL or on treatment for DM; and (3) TG ≥ 150mg/dL or HDL-C < 40 mg/dL).
[a] estimated from multilevel Cox regression (clustered by company); Model 1, adjusted for age and sex; Model 2, further adjusted for smoking status (never-smoker, former smoker, or current smoker) and pre-existing conditions of cancer, psychiatric or cardiovascular diseases (yes or no); Model 3, adjusted for alcohol consumption (< 23 or ≥ 23 g ethanol/day), duration of sleep (< 6, 6 to < 7, or ≥ 7 hours/day), overtime working hours (< 45, 45 to < 80, or ≥ 80 hours/month), occupational physical activity (mostly sitting, mostly standing or walking, fairly active) and leisure-time physical activity (< 150 or ≥ 150 min/week) plus the covariates included in the Model 2, while smoking status was replaced with smoking intensity (never-smoker, former smoker, current smoker consuming 1-10, 11-20 or ≥ 21 cigarette/day).
